# The effect of migration on social capital and depression among older adults in China

**DOI:** 10.1007/s00127-017-1439-0

**Published:** 2017-09-15

**Authors:** Qiuju Li, Xudong Zhou, Sha Ma, Minmin Jiang, Lu Li

**Affiliations:** School of Medicine, The Institute of Social and Family Medicine, Zhejiang University, 866 Yuhangtang Rd, Hangzhou, 310058 Zhejiang People’s Republic of China

**Keywords:** Migration, Elderly, Social capital, Depression, China

## Abstract

**Purpose:**

An estimated 9 million elderly people accompanied their adult children to urban areas in China, raising concerns about their social capital and mental health following re-location. The aim of this study was to examine the effect of migration on social capital and depression among this population.

**Methods:**

Multistage stratified cluster sampling was applied to recruit the migrant and urban elderly in Hangzhou from May to August, 2013. Data were collected from face-to-face interviews by trained college students using a standardized questionnaire. Social capital measurements included cognitive (generalized trust and reciprocity) and structure (support from individual and social contact) aspects. Depression was measured by Geriatric Depression Scale-30 (GDS-30). Chi-square tests and binary logistic regression models were used for analysis.

**Results:**

A total of 1248 migrant elderly and 1322 urban elderly were eligible for analysis. After adjusting for a range of confounder factors, binary logistic regression models revealed that migrant elderly reported significantly lower levels of generalized trust [OR = 1.34, 95% CI (1.10–1.64)], reciprocity [OR = 1.55, 95% CI (1.29–1.87)], support from individual [OR = 1.96, 95% CI (1.61–2.38)] and social contact [OR = 3.27, 95% CI (2.70–3.97)]. In the full adjusted model, migrant elderly were more likely to be mentally unhealthy [OR = 1.85, 95% CI (1.44–2.36)] compared with urban elderly.

**Conclusions:**

Migrant elderly suffered from a lower mental health status and social capital than their urban counterparts in the emigrating city. Attention should focus on improving the social capital and mental health of this growing population.

## Introduction

A tragedy occurred in Nanjing, Jiangsu province, in March 2016. One rural-to-urban migrant elderly committed suicide after being depressive for failing to adapt to urban life [1]. This kind of elderly health problem appearing during the process of rapid social change is exactly what this research focuses on.

According to the 2010 census, there were 221 million rural-to-urban migrants in China (Statistics of the Fifth National Census in 2010). Since the early 1990s, one of the vital changes in migration patterns is from individual to family migration [2], with young children and aging parents joining migrant workers. An estimated 6% of all migrants (approximately 9 million individuals) were aged 60 years and over as of 2010, an increase of 871,000 since 2005 [3]. Further rapid increases have been predicted during this current decade due to the one child policy and an aging population [4].

As a new social phenomenon, these elderly migrants have received considerable public and political attention in recent years. Family factors, such as looking after grandchildren, were the main migration motivation of Chinese older adults, which is different with that of other migrants [5, 6]. As older people move, they face drastic change on lifestyle and living environment, which have been reported by World Health Organization as main factors affecting health. Besides, older migrants face many challenges in adapting to the new environment, especially for the rural-to-urban migrant elderly. China’s urban–rural dualistic structure has created a dual lifestyle and cultural belief. Specifically, China’s household registration, Hukou system, which discriminates between urban residents and migrants, adds an additional welfare strain [7]. Restrictions on access to healthcare, employment, education, housing, and social insurance as local residents, leads to inequality of social capital and health. Such circumstances may increase risk of late-life depression, which has been identified as a leading cause of morbidity and mortality in older adults [8, 9].

Migration and migration-related processes have been widely found to increase risk for depression [10]. Existing literature in China has identified the huge impact of migration on the wellbeing of migrant workers and left behind elderly. The majority of studies reported that temporary rural-to-urban migrant workers have a higher risk of depression than their urban counterparts [11–14], and left behind elderly are more likely to be mentally unhealthy [15] and have lower social support [16, 17]. However, migrant elderly are less economically and physically able to overcome the negative effects of migration compared with younger migrants and may suffer more directly negative effect of migration than left behind elderly. These might seriously jeopardize the wellbeing of migrant elderly. Furthermore, researches to date focusing on the sizable elderly migrant population in China have been limited to studies concentrated on the characteristics of migration [4, 18, 19], and social adaptation [20], with no studies examining risk for depression.

Social capital—such as community involvement, trusted others, and a sense of belonging—is likely to play an important role in the adjustment to destination community for elderly migrants. Defined broadly as the features of social organization that can improve the efficacy of society by facilitating coordinated actions, social capital has been found to be strongly associated with physical and mental health among Chinese populations [21–24]. While the process of internal migration may be a threat to social capital [25], such as risks including for example social and linguistic isolation [26], robust social capital may also serve as a protective factor to well-being. Therefore, additional research is needed to clarify the relationships among social capital, depression, and migration of elderly people in the Chinese context.

In this study, we sought to explore the effect of migration on social capital and depression of migrant elderly by comparison with local urban elderly. The specific objectives of this study were to investigate whether there are differences in the prevalence of depression and social capital between migrant and urban elderly and to examine how migration-related depression might be mediated by social capital. We hypothesized that migrant elderly are more likely to be depressed and have lower social capital than urban elderly. We also expected that the relationship between migration and depression will be mediated by social capital.

## Methods

### Participants and procedures

A cross-sectional survey was conducted in Hangzhou between May and August 2013. Hangzhou is a relatively developed city in China, with an urban per capita GDP of $14,013 USD in 2013 (compared with $15,000 USD for developed countries in 2012). Migrant elderly were defined as individuals aged 60 years and over who have been living in Hangzhou for at least 3 months and do not have local residency status. Urban elderly, the counterparts of migrant elderly, were people who have been living in Hangzhou for at least 6 months and do hold local residency status.

Research participants were recruited by multistage stratified cluster sampling. For migrant elderly, two districts with high densities of immigrant populations (Yuhang and Binjiang) were selected from Hangzhou’s total 13 districts as study sites, and one sub-district was randomly selected from each of these. Two communities were then randomly selected from each sub-district giving a total of four communities. Then local community leaders were asked to provide a complete list of migrant elderly living in each community. For the urban elderly, three districts (Xihu, Gongshu, and Yuhang) were randomly selected to, respectively, represent high, middle, and low level of urbanization. One sub-district was then randomly selected from each of the three districts, and two communities randomly selected from each of these to represent high or low development level. After that, local general practitioners were asked to provide a list of the names of the urban elderly living in each community according to the health files.

Participants aged 60 years and over, matching the inclusion criteria, willing to participate, and able to communicate in Mandarin Chinese, were invited to a face-to-face interview by trained interviewers. The average length of the interview was 30 min. Those who had cognitive impairment or diagnosis of dementia were excluded from this study. All the elderly were initially approached by local community leaders or general practitioners about their willingness to complete a questionnaire of social capital and health. Those that expressed willingness to participate were asked to come to community center to complete questionnaire. Informed consent was obtained from participants and ethical approval for the study had been gained from Zhejiang University Research Ethics Committee.

### Measures

#### Individual-level Social Capital

Cognitive Social Capital was measured with by asking respondents to show extent of agreement with a series of statements about trust and reciprocity [27]. Trust in others was assessed with the following single item: “Generally speaking, can most people be trusted?”. Responses to the question ranged from ‘strongly agree’ (scored 5) to ‘strongly disagree’ (scored 1). The scores of the trust in the analysis were dichotomized as high trust (trust = 4–5) and low trust (trust = 1–3). Reciprocity was assessed by four items adapted from the World Bank Social Capital Scale [28]. These items were: “In addition to concerning themselves with their own business, are neighbors in the village also concerned about other people’s business and community matters?”, “Villagers will provide help if someone really needs it”, “I would lend money to my neighbor if he/she needs it to see a doctor”, and “I would like to support a project that might not benefit me most, but benefit other villagers”. The validity and reliability of each question or statement have been assessed in China [27]. Response to each question ranged from ‘strongly agree’ (scored 5) to ‘strongly disagree’ (scored 1). Then the scores were summed up and dichotomized as high reciprocity if the sum score is at or above the mean and low reciprocity if otherwise.

Structural social capital was measured with items concerning social support and social contact. Social support from individuals was measured by the question “In the last 12 months, have you received any help or support that is emotional help, economic help or assistance in helping you know or do things?” Participants then indicated ‘yes’ (support received) or ‘no’ (support not received) for a range of sources (Family, Friends who are not neighbors, neighbors, government officials/civil service, community leaders, charitable organizations/NGO, religious leaders, politicians, and Other). Each source of help received generated a sum score which was categorized in the analysis as high support received (if sum score ≥1), or low support received (responding to score 0). Social contact was assessed by the question “On average, how many times a week do you and others (family members, neighbors, friends, etc.) usually drop in on one another?” The answer was categorized as high social contact (if responding ≥1) and low social contact (if responding to 0).

#### Depression

Depressive symptoms were measured by the 30-item Geriatric Depression Scale (GDS-30), which has been widely used for the elderly worldwide [29]. The validity and reliability of GDS-30 have been extensively assessed in China [30, 31]. Respondents were asked 30 yes/no questions relating to symptoms of depression and anxiety experienced during the previous week. The sum score ranged from 0 to 30. Those who reported 10 or fewer symptoms were considered normal, 11–20 symptoms were mildly depressed, and 21 or more symptoms were moderately to severely depressed [32]. The Cronbach’s alpha coefficient of the GDS-30 was 0.86 for the entire sample, 0.84 for urban sample and 0.88 for migrant sample.

#### Demographic characteristics

Demographic characteristics, which were treated as covariates, included sex (Female, Male), age group (60–69, 70–79, 80-years), marital status (Married, Widowed, Other), living arrangement (Living with family member, Living alone), religious faith (Having, Not having) and physical function. Education level was categorized into “illiterate, primary school, junior high school, and senior high school and high”. Annual household income (RMB) was divided into groups of 0–24,999, 25,000–49,999, 50,000–74,999, and 75,000 or more.

### Statistical analysis

Chi-square tests (for categorical variables) or ANOVA (for continuous variables) were first used to examine the difference of demographic variables, social capital, and depression among the two groups (migrant or urban). Binary logistic regression models were then conducted to investigate the difference in social capital and depression between the two elderly groups after adjusting for a range of potential cofounders, such as sex, age, educational level, marital status, living arrangement and others. Depression was tested according to three different models. The variable sets were entered stepwise in the following sequence: (1) sex, age, and migrant status; (2) educational level, marital status, household income, living arrangement, religious faith and physical function; and (3) support from individual, social contact, generalized trust and reciprocity. The odds ratios (OR), 95% confidence intervals (CI), and *p* value were presented. Statistical significance was set at a *p* value of less than 0.05. All statistical analyses were performed using SPSS for Windows Version 23.0.

## Results

### Sample characteristics

A total of 1378 migrant elderly and 1343 urban elderly participated in the study, of which 1248 (90.6%), and 1322 (98.4%) in each group, respectively, were eligible for analysis. Table 1 presents the full descriptive statistics of the study participants. Chi-square tests revealed notable disparities between two elderly groups. The age of the migrant elderly ranged from 60 to 90 years, with the majority (80.7%) being aged 60–69 years. They were significantly younger than local urban elderly (*p* = 0.000). Migrant elderly were more likely than urban elderly to be male (46.5% compared with 40.1%, *p* = 0.001), married (82.6% compared with 77.6%, *p* = 0.000), highly educated (57.2% for junior high school or more, compared with 46.3%, *p* = 0.000), live with family member (93.8% compared with 68.7%, *p* = 0.000), and have better physical function (89.0 compared with 81.7, *p* = 0.000). More than 70% of migrants’ annual household income was less than 50,000 RMB Yuan (About 8250 USD at the time of survey), poorer than urban elderly (*p* = 0.000).

**Table 1:** Socio-demographic characteristics of the participants [*N* (%)]

Variable	Urban elderly (*N* = 1322)	Migrant elderly (*N* = 1248)	*χ* ^2^ or *t*	*p*
Sex			10.662	0.001
Male	530 (40.1)	580 (46.5)		
Female	792 (59.9)	668 (53.5)		
Age			211.459	0.000
60–69	748 (56.6)	1007 (80.7)		
70–79	356 (26.9)	208 (16.7)		
80–	218 (16.5)	33 (2.6)		
Marital status			23.352	0.000
Married	1015 (77.6)	1031 (82.6)		
Widowed	264 (20.2)	170 (13.6)		
Other	29 (2.2)	47 (3.8)		
Education level			56.321	0.000
Illiteracy	350 (26.6)	193 (15.5)		
Primary school	357 (27.1)	341 (27.3)		
Junior high school	337 (25.6)	354 (28.4)		
Senior high school and high	273 (20.7)	360 (28.8)		
Annual household income (RMB)			124.191	0.000
0–24,999	294 (22.2)	406 (32.5)		
25,000–49,999	356 (26.9)	474 (38.0)		
50,000–74,999	457 (34.6)	231 (18.5)		
75,000–	215 (16.3)	137 (11.0)		
Living arrangement			255.741	0.000
Living with family member	908 (68.7)	1141 (93.8)		
Living alone	414 (31.3)	76 (6.2)		
Religious faith			0.050	0.823
Having	376 (28.4)	350 (28.0)		
Not having	946 (71.6)	898 (72.0)		
Physical function (mean ± SD)	81.7 ± 21.1	89.0 ± 16.7	−9.811	0.000
Generalized trust			4.990	0.025
Low	910 (69.0)	911 (73.0)		
High	409 (31.0)	337 (27.0)		
Reciprocity			7.905	0.005
Low (less than the mean)	572 (43.3)	609 (48.8)		
High (above the mean)	750 (56.7)	639 (51.2)		
Support from individual			42.756	0.000
Low (sum score = 0)	378 (28.6)	510 (40.9)		
High (sum score ≥1)	944 (71.4)	738 (59.1)		
Social contact			189.145	0.000
Low (less than once a week)	563 (42.6)	868 (69.6)		
High (at least once a week)	759 (57.4)	380 (30.4)		
Depression			13.225	0.000
No depression (0–10)	995 (75.3)	859 (68.8)		
Mild-to-severe depression (11–30)	327 (24.7)	389 (31.2)		

In terms of migration, more than half of the migrant elderly (56.3%) had moved from urban to urban areas, with the remaining 43.8% having moved from rural to urban areas. About 43% of the migrant elderly had stayed in Hangzhou for less than or equal to 3 years. 44.7% of migrants came from other provinces, with the other 55.3% coming from within Zhejiang province. Nearly 87.6% reported that they had moved to Hangzhou to take care of grandchildren (Table 2).

**Table 2:** Patterns of migration for migrant elderly

Variable	*N* (%)
Household registration	
Urban	702 (56.3)
Rural	546 (43.8)
Hometown	
Zhejiang province	690 (55.3)
Other province	558 (44.7)
Length of staying in Hangzhou (years)	
≤3	537 (43.0)
>3 and ≤6	370 (29.6)
>6	341 (27.3)
Reason of moving to Hangzhou	
Taking care of child	628 (52.6)
Taking care of grandchildren	1045 (87.6)
Relying on child	160 (13.4)
Working	34 (2.8)
Other	23 (1.9)

### Social capital

Compared with urban elderly, a significantly more migrant elderly reported lower generalized trust (73.0 vs. 69.0%, *p* = 0.025), lower reciprocity (48.8 vs. 43.3%, *p* = 0.005), lower support from individual (40.9 vs. 28.6%, *p* = 0.000) and lower social contact (69.6 vs. 42.6%, *p* = 0000) (Table 1). After adjusting for sex, age, annual household income, education level, marital status, living arrangement, religion faith and physical function, migrant elderly still reported significantly lower levels of generalized trust [OR = 1.34, 95% CI (1.10–1.64)], reciprocity [OR = 1.55, 95% CI (1.29–1.87)], support from individual [OR = 1.96, 95% CI (1.61–2.38)], and social contact [OR = 3.27, 95% CI (2.70–3.97)] (Table 3).

**Table 3:** Odd ratios (OR) with 95% confidence intervals (CI) of poor social capital indicators

Variable	Generalized trust		Reciprocity		Support from individual		Social contact	
	OR (95% CI)	*p*	OR (95% CI)	*p*	OR (95% CI)	*p*	OR (95% CI)	*p*
Migrant status								
Urban	1		1		1		1	
Migrant	1.34 (1.10–1.64)	0.004	1.55 (1.29–1.87)	0.000	1.96 (1.61–2.38)	0.000	3.27 (2.70–3.97)	0.000
Sex								
Female	1		1		1		1	
Male	1.32 (1.09–1.59)	0.004	1.24 (1.04–1.47)	0.015	1.07 (0.90–1.28)	0.437	0.71 (0.60–0.85)	0.000
Age								
60–69	1		1		1		1	
70–79	0.73 (0.58–0.91)	0.006	1.00 (0.81–1.24)	0.999	1.34 (1.08–1.66)	0.009	1.25 (1.01–1.56)	0.044
80–	0.50 (0.36–0.70)	0.000	0.83 (0.60–1.14)	0.251	0.96 (0.69–1.35)	0.830	1.82 (1.31–2.51)	0.000
Annual household income (RMB)								
75,000–	1		1		1		1	
50,000–74,999	0.93 (0.70–1.25)	0.635	0.84 (0.64–1.11)	0.216	0.95 (0.72–1.26)	0.740	1.59 (1.20–2.10)	0.001
25,000–49,999	0.97 (0.72–1.31)	0.853	0.83 (0.63–1.10)	0.193	0.80 (0.60–1.07)	0.133	0.92 (0.69–1.22)	0.553
0–24,999	0.97 (0.70–1.34)	0.832	1.06 (0.78–1.42)	0.721	0.77 (0.57–1.05)	0.099	0.96 (0.71–1.31)	0.806
Education level								
Senior high school and high 1			1		1		1	
Junior high school	0.84 (0.66–1.07)	0.167	0.97 (0.77–1.22)	0.777	1.24 (0.98–1.58)	0.078	1.25 (0.99–1.59)	0.063
Primary school	1.18 (0.91–1.53)	0.218	1.52 (1.20–1.93)	0.001	1.17 (0.91–1.51)	0.213	1.21 (0.95–1.56)	0.123
Illiteracy	1.64 (1.21–2.23)	0.002	2.48 (1.88–3.26)	0.000	1.60 (1.21–2.12)	0.001	0.75 (0.57–1.00)	0.047
Marital status								
Married	1		1		1		1	
Widowed	0.73 (0.56–0.96)	0.022	0.77 (0.60–0.99)	0.045	1.22 (0.95–1.58)	0.120	1.37 (1.06–1.78)	0.017
Other	0.73 (0.43–1.22)	0.226	1.00 (0.62–1.63)	0.998	1.59 (0.99–2.57)	0.056	1.67 (0.98–2.85)	0.058
Living arrangement								
Living with family member 1			1		1		1	
Living alone	1.51 (1.16–1.96)	0.002	1.51 (1.19–1.90)	0.001	1.14 (0.90–1.45)	0.276	0.54 (0.43–0.69)	0.000
Religious faith								
Having	1		1		1		1	
Not having	1.01 (0.82–1.23)	0.962	1.09 (0.91–1.31)	0.364	1.05 (0.87–1.28)	0.587	0.86 (0.71–1.04)	0.129
Physical function	0.99 (0.98–0.99)	0.000	0.99 (0.98–0.99)	0.000	1.00 (1.00–1.01)	0.451	1.00 (1.00–1.01)	0.646

Compared with urban-to-urban migrant elderly, rural-to-urban migrant elderly demonstrated lower reciprocity (54.8 vs. 44.2%, *p* = 0.000), lower support from individual (45.2 vs. 37.5%, *p* = 0.006) and lower social contact (74.2 vs. 66.0%, *p* = 0002), with no significant difference reported in generalized trust (Table 4). After adjusting for sex and age, rural-to-urban migrant elderly were still significantly more likely to have low reciprocity [OR = 1.59, 95% CI (1.26–1.99)], low support from individual [OR = 1.49, 95% CI (1.18–1.88)], and low social contact [OR = 1.50, 95% CI (1.17–1.93)].

**Table 4:** Social capital and depression of rural-to-urban and urban-to-urban migrant elderly *N* (%)

Variable	Urban-to-urban migrant elderly (*N* = 702)	Rural-to-urban migrant elderly (*N* = 546)	*χ* ^2^ or *t*	*p*
Generalized trust				
Low	509 (72.5)	402 (73.6)	0.195	0.659
High	193 (27.5)	144 (26.4)		
Reciprocity			13.818	0.000
Low (less than the mean)	310 (44.2)	299 (54.8)		
High (above the mean)	392 (55.8)	247 (45.2)		
Support from individual			7.680	0.006
Low (sum score = 0)	263 (37.5)	247 (45.2)		
High (sum score ≥1)	439 (62.5)	299 (54.8)		
Social contact			9.802	0.002
Low (less than once a week)	463 (66.0)	405 (74.2)		
High (at least once a week)	239 (34.0)	141 (25.8)		
Depression			8.606	0.003
No depression (0–10)	507 (72.2)	352 (64.5)		
Mild-to-severe depression (11–30)	195 (27.8)	194 (35.5)		

### Depression

The prevalence of depressive symptoms was 31.2% in migrant elderly and 24.7% in urban elderly (*χ* ^2^ = 13.225, *p* = 0.000) (Table 1). Results of logistic regressions on depression are presented in Table 5. In the crude model, which adjusted for age and sex only, migrant elderly demonstrated higher depression levels than urban elderly [OR = 1.78, 95% CI (1.47–2.15)]. However, in the fully adjusted Model 3, the effect of migration was attenuated from 2.11 to 1.85 by the inclusion of social capital indicators in Model 2, suggesting that in a small part, some depression disadvantage was caused by lower cognitive social capital (generalized trust and reciprocity) rather than structure social capital (support from individual and social contact). Being younger, married, having higher educational level, higher annual household income and better physical function were also associated with better psychological health.

**Table 5:** Odd ratios (OR) with 95% confidence intervals (CI) of poor depression for elderly

Variable	Model 1		Model 2		Model 3	
	OR (95% CI)	*p*	OR (95% CI)	*p*	OR (95% CI)	*p*
Migrant status						
Urban	1		1		1	
Migrant	1.78 (1.47–2.15)	0.000	2.11 (1.68–2.65)	0.000	1.85 (1.44–2.36)	0.000
Sex						
Female	1		1		1	
Male	0.97 (0.81–1.16)	0.721	1.23 (1.01–1.50)	0.040	1.14 (0.93–1.40)	0.208
Age						
60–69	1		1		1	
70–79	2.10 (1.69–2.60)	0.000	1.50 (1.18–1.90)	0.001	1.55 (1.21–1.99)	0.001
80–	3.08 (2.29–4.13)	0.000	1.24 (0.87–1.76)	0.229	1.42 (0.98–2.05)	0.062
Annual household income (RMB)						
75,000–			1		1	
50,000–74,999			1.12 (0.79–1.59)	0.528	1.17 (0.81–1.68)	0.397
25,000–49,999			1.32 (0.94–1.86)	0.107	1.40 (0.99–2.00)	0.060
0–24999			1.49 (1.04–2.14)	0.029	1.54 (1.06–2.22)	0.024
Education level						
Senior high school and high			1		1	
Junior high school			0.90 (0.67–1.20)	0.462	0.92 (0.68–1.24)	0.571
Primary school			1.51 (1.14–2.00)	0.004	1.40 (1.04–1.88)	0.026
Illiteracy			2.42 (1.77–3.30)	0.000	1.98 (1.43–2.73)	0.000
Marital status			1		1	
Married			1		1	
Widowed			1.35 (1.03–1.76)	0.031	1.51 (1.14–2.00)	0.004
Other			2.09 (1.26–3.46)	0.004	2.24 (1.30–3.86)	0.004
Living arrangement						
Living with family member			1		1	
Living alone			1.04 (0.80–1.36)	0.774	0.88 (0.67–1.16)	0.366
Religious faith						
Having			1		1	
Not having			0.91 (0.73–1.12)	0.372	0.91 (0.73–1.13)	0.383
Physical function			0.97 (0.97–0.98)	0.000	0.98 (0.97–0.98)	0.000
Generalized trust						
High					1	
Low					2.14 (1.65–2.76)	0.000
Reciprocity						
High					1	
Low					2.46 (2.00–3.03)	0.000
Support from individual						
High					1	
Low					1.19 (0.97–1.47)	0.103
Social contact						
High					1	
Low					0.99 (0.80–1.23)	0.930

The prevalence of depression was 27.8% in urban-to-urban migrant elderly and 35.5% in rural-to-urban migrant elderly (*χ* ^2^ = 8.606, *p* = 0.003) (Table 4). After adjusting for sex and age, rural-to-urban migrant elderly were still more likely to have higher depression [OR = 1.63, 95% CI (1.27–2.10)].

## Discussion

### Summary

To the best of our knowledge, this is the first study to evaluate the relationships among migration and depression and social capital among elderly in China. Our study showed that after controlling for the confounding factors, migrant elderly are more likely to have lower social capital and higher depression compared with urban elderly in Hangzhou where they had immigrated, and rural-to-urban migrant elderly have worse social capital and depression than urban-to-urban migrant elderly. Moreover, the relationship between migration and depression is mediated by cognitive social capital (trust and reciprocity).

### Contribution to existing literature

First, the characteristics of old migrants may reflect the migration selectivity of Chinese older adults. Majority of them migrate to look after grandchildren, which is a prevalent phenomenon in the modern Chinese society. In many other countries, older migrants tend to migrate for better medical care, being nearer family and friends, or better environment [33]. Besides, people with better educational attainment are more inclined toward migration. One reason is that high-educated people know more about the outside world and have a greater interest in gaining experience of it [3]. The other one is that rural people, especially those with lower education, are more influenced by the traditional cultural belief that one should die in the place where they were born, while urban or well-educated residents are less such affected [34].

Second, this study demonstrated that migrant elderly possess less social capital compared with urban elderly. Emigration to a new place results in loss of network and social interaction from the place of origin. Majority of migrant elderly in our study have short migration duration, which has negative effect on integration and adaptation [35].They may lack resources and infrastructure that can alleviate their acculturation and rebuilt their social network [36]. Furthermore, nearly half of old migrants came from rural areas or other provinces. They would have even poorer capacity to adapt to urban life with new social norms, values, and customs, and overcome cultural barriers such as different dialects, which may reduce the ability to maintain and develop social capital [37]. Besides, stigma against migrants as a common component of social discrimination, which significantly affects social capital reconstruction [38], has been documented in many studies [39, 40].

Third, the finding of more depression among migrant elderly is in keeping with studies from recent meta-analytic review [41], Shenzhen [11] and Beijing [42]. In contrast, studies in Guangzhou [43] and Hangzhou [44] found migrant workers had better mental wellbeing than local urban counterparts had. Besides, some studies, such as the ones in Beijing [45] and Peru [46], showed similar level of mental health in the two groups.

There might be several possible explanations for higher rate of depression among migration elderly in this study. First, the majority of them spent much time looking after grandchildren and doing housework, describing themselves as free nannies. Their adult children are usually busy at work and have little time accompanying and talking with their migrant elderly parents. Also, it is likely to have a conflict with adult children on how to raise grandchildren and on different lifestyle. Lacking emotional support from adult children and the proneness of family conflict are associated with higher risk for depression [47, 48]. Second, with the reduction of family size and the weakening of family function, non family-based network and resources become more important for the elderly. The lack of social support from friends in the original place is positively associated with depression among Chinese elderly [48, 49]. Third, health inequalities may stem from difficulties in accessing resources, as migrants have demonstrated restricted access to health services and social welfare [50, 51]. While efforts to address health disparities, such as the occurrence of a new health insurance system, are underway in China, existing services and programs have not been fully utilized by migrants yet.

Finally, our results support the finding that cognitive social capital is more important than structural social capital as protective factors of depression. Within psychological health studies, cognitive social capital, i.e., trust and reciprocity, seems to have a stronger impact on health than structural aspects [21, 52, 53]. While the structural aspects [54] provide support through formal and informal institutions, cognitive social capital may increase sense of belongingness within and between communities, which would be beneficial, particularly with regard to mental health.

In view of the massive numbers of migrant elderly in China, this problem is rather alarming and warrants close attention from the Chinese authorities. More social services should be provided to create a friendly community environment, which can not only reduce urban–rural barriers, but also help migrant elderly rebuild social capital quickly, thereby improving the wellbeing. Besides, related childcare policy can be proposed to support young families, prevent some involuntary migration happening and relieve the migrant elderly’s pressure on childcare.

### Strengths and limitations

The current study has several limitations. First, the cross-sectional study was unable to infer any causal relationship between migration, depression, and social capital. Future longitudinal research should be designed to help further understand any causation. Second, our comparison group only included urban elderly at the place of destination; the rural elderly from the place of origin would provide a further and worthwhile comparison group. Third, this study only discussed social capital at an individual level. Future studies might add community level social capital. Last, while the sample size is large, it is taken from a relatively affluent large city in eastern China, where migrant elderly may enjoy relatively high living conditions and life satisfaction. Thus, it is not supposed to extrapolate the findings to the whole country.

## Conclusions

Our study suggests that migrant elderly might experience higher prevalence of depression and lower level of social capital than urban elderly groups. The depression disadvantage is partly accounted for by lower level of cognitive social capital (trust and reciprocity). In view of the dynamic characteristics of migration, longitudinal studies with representative samples are needed to help us better understand the etiology of mental health problem and changing process of social capital among Chinese migrant elderly.
